# MOSMAP: Mosquito metagenome analysis pipeline

**DOI:** 10.6026/973206300210110

**Published:** 2025-02-28

**Authors:** Umay Kulsum, Chitra Patankar, Debasis Biswas

**Affiliations:** 1 Department of Microbiology, All India Institute of Medical Sciences, Bhopal, India

**Keywords:** Mosquito metagenome analysis (MosMAP), bioinformatics pipeline, taxonomic classification, species abundance estimation

## Abstract

MosMAP is a bioinformatics pipeline designed for mosquito metagenome analysis. MosMAP automates essential processes like quality control, taxonomic classification, species abundance estimation and visualization by integrating tools such as Trimgalore, Kraken 2, Bracken and Krona into a user-friendly workflow. Each of these tools is integrated to ensure a smooth and efficient workflow from raw data to interpretable results. The pipeline simplifies complex bioinformatics tasks, making them accessible to researchers with limited computational expertise. MosMAP demonstrated high concordance with standard bioinformatics workflows such as Kraken and Bracken in terms of read retention, taxonomic accuracy and abundance estimation when applied to metagenomes of mosquito collected in Bhopal, India. This accessible pipeline promotes the simplification of meta-genomics, supporting research in microbiology, ecology and vector-borne diseases.

## Background:

Metagenomic analysis has become an indispensable tool for exploring the diversity of microbial and viral communities in environmental, clinical and biological samples [1]. The advent of Next-Generation Sequencing (NGS) technologies has significantly enhanced our knowledge of genomic data, providing deeper insights into the structure and function of microbial and viral populations [2]. However, the process of analysing metagenomic data is often complicated by the large volume of sequencing reads, the diversity of organisms present and the necessity for multiple bioinformatics tools to process, classify and visualize the data [3].

Tools like *SqueezeMeta* and MOCAT2 have attempted to ease Metagenomic workflows that often require manual configuration of multiple bioinformatics tools, making the process time-consuming and technically demanding [4, 5]. Therefore, it is of interest to present MosMAP, an integrated and automated pipeline designed to streamline mosquito metagenome analysis. MosMAP combines several established bioinformatics tools into a single, user-friendly shell script that performs essential tasks such as sequence quality filtering, taxonomic classification, viral species abundance estimation and interactive data visualization without the need for extensive computational expertise.

## Input:

MosMAP accepts raw sequencing data in FASTQ format, either compressed or uncompressed. The pipeline requires a simple text file (list.txt) containing the names of the input FASTQ files. Users must install all the prerequisites including the MiniKraken database for taxonomic classification by running the *install.sh* script and then execute *pipeline.sh*to process and analyse the data. Additionally, the user must specify the path to their Anaconda installation, as several of the tools utilized in MosMAP are distributed via Anaconda. MosMAP is hosted on Git Hub, with detailed instructions available in the README file. The minimal hardware requirement for running MosMAP is 8 GB of RAM, making it suitable for most standard computational setups.

## Output:

## MosMAP generates a series of outputs, including:

[1] Quality control reports: Trimgalore and FastQC outputs that summarize read quality and trimming statistics.

[2] Taxonomic classification reports: Kraken2 outputs detailing taxonomic assignments of sequencing reads.

[3] Abundance estimation: Bracken-generated tables that provide taxonomic composition.

[4] Interactive visualization: Krona plots in HTML format that allow users to explore taxonomic data interactively.

[5] Combined report: A combined report summarizing the abundance estimations across all samples is produced.

## Performance highlights:

Tools like Trimgalore ensure high-quality sequence data by trimming low-quality bases and adapter sequences [6], while FastQC provides comprehensive quality control reports [7]. The effectiveness of these pre-processing steps in MosMAP was evaluated by comparing read retention rates after quality control. The results showed a high level of concordance between MosMAP and the standard tool, indicating that MosMAP's pre-processing methods effectively retained high-quality reads while removing low-quality sequences and adapters. Kraken, integrated within MosMAP, was used to perform taxonomic classification of the metagenome reads [8]. The classification results were compared with those obtained from Kraken run individually, revealing a strong agreement in the identification of taxa. This consistency underscores the reliability of MosMAP for taxonomic identification in mosquito metagenomes.

The relative abundance of taxa estimated by MosMAP was compared to estimates from standard pipeline Bracken, which complements Kraken by refining abundance estimates and providing detailed insights into the microbial community structure [9]. This comparison showed that MosMAP's abundance estimation was highly consistent with those from Bracken run individually, providing accurate and reliable abundance profiles for the mosquito metagenome samples. The integration of Krona for visualization facilitates the exploration of metagenomic data through interactive charts [10]. By developing a bash script that integrates all these standard tools, we have demonstrated that MosMAP can generate results that are highly consistent with those obtained from running the tools individually. MosMAP, thus, advances scientific research on mosquito Metagenomic analysis by consolidating multiple bioinformatics tools into a single, stream-lined, automated workflow that ensures high concordance with standard methodologies and significantly reduces analysis time and technical barriers. Furthermore, its potential for integration with expanded taxonomic databases and functional annotation tools makes it a scalable resource for facilitating large microbial and viral surveillance studies in mosquito populations and thus contributing to research on vector-borne diseases.

## Caveats:

Despite being impactful in improving accessibility to bioinformatics analysis, we understand the current version of MosMAP has sufficient scope for further improvement. Running the analysis on the standard Kraken database instead of MiniKraken, will enable the discovery of novel genetic variants and emerging viral strains. Secondly, the functional annotation of the identified genes, if enabled in this pipeline will further enrich the knowledge output generated from the Metagenomic analysis, providing deeper insights and advancing our understanding in this field.

## Future development:

The future development of MosMAP will focus on incorporating the standard Kraken database to improve taxonomic classification accuracy and the identification of novel species. Additionally, efforts will be made toward developing a user-friendly graphical user interface (GUI) to make the pipeline even more accessible to researchers without command-line experience. These developments will ensure that MosMAP remains a robust and versatile tool for the metagenomics community.

## Conclusion:

MosMAP is a user-friendly bioinformatics pipeline that streamlines mosquito metagenome analysis by integrating essential tools for quality control, taxonomic classification, abundance estimation and visualization. By enhancing accessibility and efficiency, MosMAP has the potential to advance research in microbial ecology, vector-borne diseases and environmental microbiology.

## Availability and requirements:

## Project home page:

https://github.com/Biomedinformatics/MosMAP

## Operating system(s):

Linux

## Programming language:

Bash script

## Other requirements:

Anaconda

## Ethics approval and consent to participate:

Not Applicable

## Consent for publication:

Not Applicable

## Availability of data and materials:

Not Applicable

## Competing interests:

The author(s) declare no competing interests.

## Funding:

None

## Authors' contributions:

The conceptualization of the study was carried out by UK, CP and DB. UK developed the methodology, while data curation was handled by CP and DB. The formal analysis was conducted by UK, CP and DB. The original draft of the manuscript was written by UK and DB and CP and DB reviewed and edited the draft. The project was administered and supervised by DB.
